# Supplementation with Folic Acid or 5-Methyltetrahydrofolate and Prevention of Neural Tube Defects: An Evidence-Based Narrative Review

**DOI:** 10.3390/nu16183154

**Published:** 2024-09-18

**Authors:** María de Lourdes Samaniego-Vaesken, Carmen Morais-Moreno, Alejandra Carretero-Krug, Ana María Puga, Ana María Montero-Bravo, Teresa Partearroyo, Varela-Moreiras Gregorio

**Affiliations:** 1 Grupo USP-CEU de Excelencia “Nutrición para la vida (Nutrition for Life)”, Ref: E02/0720, Departamento de Ciencias Farmacéuticas y de la Salud, Facultad de Farmacia, Universidad San Pablo-CEU, CEU Universities, Urbanización Montepríncipe, 28660 Boadilla del Monte, Spain; l.samaniego@ceu.es (M.d.L.S.-V.); carmen.moraismoreno@ceu.es (C.M.-M.); alejandra.carreterokrug@ceu.es (A.C.-K.); anamaria.pugagimenezazca@ceu.es (A.M.P.); amontero.fcex@ceu.es (A.M.M.-B.); t.partearroyo@ceu.es (T.P.); 2 Instituto Universitario CEU Alimentación y Sociedad, Facultad de Farmacia, Universidad San Pablo-CEU, CEU Universities, Urbanización Montepríncipe, 28660 Boadilla del Monte, Spain

**Keywords:** folic acid, folates, homocysteine, 5-methyltetrahydrofolate, fortification, supplementation, pregnancy, neural tube defects, NTD

## Abstract

**Background:** Folic acid (FA), which in its chemical form is pteroylglutamic acid, is the fully oxidised, water-soluble, monoglutamic form of vitamin B9. This compound is part of the folate group but with higher bioavailability, and it is found in vitamin supplements and fortified foods and drugs. Folate metabolism is complex and associated with various metabolic pathways, all of which confer protection on the cell and allow its survival. **Methods**: We conducted a non-systematic search of articles published in English and Spanish including controlled trials, cohort studies, systematic re-views, and meta-analyses were included, as well as key studies in animal models related to pharmacokinetic studies. Search terms encompassed: “folic acid”, “folates”, “5-metyltetrahydrofolate”, “5-MTHF”, “neural tube defects”, “supplementation”, “fortification”, AND “homocysteine” **Results**: A crucial role demonstrated for FA is to help prevent neural tube defects (NTDs). However, more studies are definitely still needed to establish 5-MTHF as a safe and effective therapeutic approach comparable with FA. Moreover, there is a lack of clinical studies that evaluate the efficacy of 5-MTHF supplementation in the prevention of NTDs. The present evidence-based narrative review discusses differences between FA and 5-MTHF in terms of structure, metabolism, bioavailability, clinical efficacy, and safety. **Conclusions**: Despite the potential value of 5-MTHF as an alternative to FA, clinical studies would be urgently needed to support the efficacy, dosage, timing, and/or safety of its use as a supplement.

## 1. Introduction

**Figure 1:**
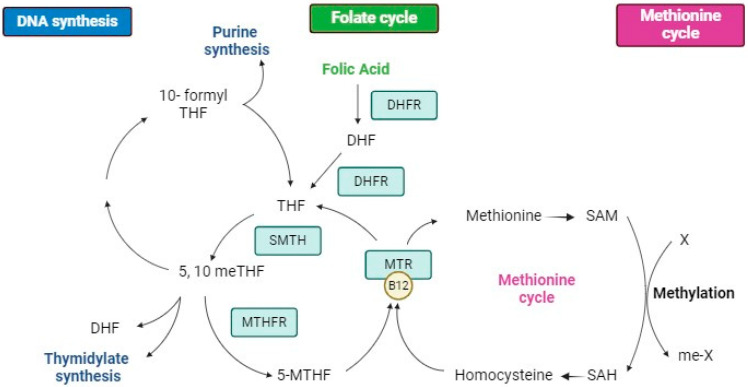
Methionine and folate cycles and connecting pathways. Created with BioRender.com.

Folate is a general term used to describe natural and synthetic forms of vitamin B9. It is a generic term for a family of compounds known as vitamers which consist of a common biochemical structure but differ in the number of glutamic acids, oxidation state, and the presence of one-carbon groups (e.g., folic acid (FA), 5-methyltetrahydrofolate (5-MTHF), tetrahydrofolate (THF), or dihydrofolate (DHF)) [1]. Folates are essential nutrients that cannot be synthesised and, therefore, must be provided through diet and/or dietary supplements [2]. Specifically, FA, whose chemical form is pteroylglutamic acid, is a synthetic compound (as is any form used for exogenous folate supplementation) with a similar folate structure but with much higher bioavailability. It is the only folate form authorised in fortified foods and, particularly, in drugs. FA must first be reduced by the dihydrofolate reductase (DHFR) enzyme in the liver to DHF, then to THF, and then to be further converted to 5,10-methylene-THF and 5-MTHF, all metabolites of the folate cycle (Figure 1). It should be noted that these metabolites are continuously interconverted to each other, given the cyclic nature of folate metabolism.

Folates are involved in two major functions: the synthesis and repair of nucleic acids and the synthesis of the amino acid methionine from homocysteine (Hcy). It is well known that Hcy accumulation in the body (so-called hyperhomocysteinemia) is associated with congenital defects in the fetus as well as chronic diseases during adulthood [3]. Furthermore, it is corroborated that FA consumption, before and during pregnancy, is beneficial in the prevention of neural tube defects (NTDs), the most common congenital anomaly, and it may also provide protection against others [4].

Several national and international agencies have established recommendations either for folate daily intake (μg/day) or supplementation, including at the international level the European Food Safety Authority (EFSA) [5], the World Health Organisation (WHO) [6], the U.S. Institute of Medicine of the National Academy of Sciences (IOM) [7], and the Centers for Disease Control and Prevention [8]. At the Spanish level, this includes Moreiras et al. in the Food Composition Tables [9], the Spanish Society of Gynecology and Obstetrics (SEGO) [10], and the Spanish Ministry of Health [11] (Table 1). Currently, despite widespread evidence of the crucial role of periconceptional supplementation, recommendations vary even for women of reproductive age and those planning pregnancy [3]. However, there is a general consensus for pregnant women, where national and international guidelines agree in recommending 400 μg/day of FA supplementation. Concerning Tolerable Upper-Intake Levels (ULs), there is a general agreement in the establishment of the ULs for FA at 1000 μg/day for adults, including pregnant and lactating women [3].

Despite the importance of including in the diet sufficient amounts of FA, especially during periods of rapid cell division and growth, data from the ANIBES (Antropometría, Ingesta y Balance Energético en España) study, representative of the Spanish population, revealed an average FA consumption of 156.3 μg/day. Thus, the proportion of adequacy for folates in the total population of women was merely 3.0% (percentage of women with FA consumption above 80% of the Reference Daily Intake) [12]. Other studies have shown similar findings: for example, a national survey of the German population showed a mean folate intake of 200 μg/day [3]. To evaluate folate status, several serum markers can be used, including red blood cell (RBC) folate, total Hcy, and serum folate. The latter is preferred over RBC-folate due to its fast response, even after supplementation, and its ability to reflect the amount available for transfer across the placenta [3].

Folate deficiency is linked to factors such as restrictive diets, culinary processes (including high temperatures), greater physiological demand (e.g., during pregnancy and breastfeeding), or increased physical activity. Other causes of folate insufficiency are vitamin-B12 deficiency (a cofactor in folate metabolism), poor intestinal absorption (damage of the villi or pH alterations, celiac or Crohn’s disease, ulcers, etc.), diseases with loss of nutrients (hepatic, renal, or anaemia), drug interactions (for example with metformin, methotrexate, acetylsalicylic acid, sulphonamides, antacids, barbiturates, and/or oral contraceptives), or even genetics (mutations in genes related to folate metabolism or transportation) [13]. In addition, there are other factors that have been hypothesised to determine the metabolic insufficiency of folates [14], including their bioavailability and bioconversion. (a) Folate bioavailability refers to the fraction that is absorbed and used in metabolic processes: dietary folates have a bioavailability of around 50%, lower than that of fortified products (close to 85%), and this, in turn, is lower than that of FA supplements, which is close to 100%. Folate bioconversion, in turn, relates to folate metabolisation, which is affected by polymorphisms in the folate cycle enzymes, such as methylenetetrahydrofolate reductase (MTHFR). These latter aspects will be discussed throughout this review.

**Table 1:** Folate intake (μg/day) and supplementation recommendations by different international and Spanish organisations.

Organisation	EFSA [15,16]	WHO [6,17]	IOM [18,19]	CDC [8]	Moreiras et al. [9]	SEGO [10]
Women of reproductive age	330 DFE	400 DFE	400 DFE	400 DFE	400 DFE	No specific recommendations
Women planning pregnancy	FA supplementation: 400 **	FA supplementation: 400	FA supplementation: 400	FA supplementation: 400	No specific recommendations	FA supplementation: 400 *
Pregnant women	FA supplementation: 400	FA supplementation: 400	FA supplementation: 400	FA supplementation: 400	600 DFE	FA supplementation: 400 *
Lactating women	500 DFE	500 DFE	500 DFE	500 DFE	500 DFE	FA supplementation: 400

EFSA: European Food Safety Authority; WHO: World Health Organisation; IOM: U.S. Institute of Medicine of the National Academy of Sciences; CDC: Centers for Disease Control and Prevention; SEGO: Sociedad Española de Ginecología y Obstetricia. DFE: dietary folate equivalents. For combined intakes of food folate and folic acid, DFEs can be computed as follows: μg DFE = μg food folate + (1.7× μg folic acid). If supplementation is specifically recommended, this figure is included instead of DFE. * Also considered other folate forms, such as 5-THF or 5-MTHF. ** 400 μg/day supplemental folate should be consumed daily for at least one month before and up to three months after conception.

FA status has been recognised as a public health challenge by many authors in the past decades [3,20]. To manage this issue, in 1998, the Food and Nutrition Board of the National Academy of the IOM recommended that all women who might become pregnant take folic acid-rich foods as part of a varied diet, in addition to taking FA supplementation. That same year, the U.S. Food and Drug Administration (FDA) required manufacturers to add FA to all grain-based food products [21]. This strategy, known as mandatory fortification, is an interesting public health strategy for the prevention of NTDs for three main reasons: (a) about half of all pregnancies are unplanned; (b) prevention of NTDs can only be effective before and during the first weeks of pregnancy, often before women know they are expecting; and (c) it avoids non-compliance with pharmacological/supplementation regimens [22]. This cost-effective measure, which has saved many lives in countries such as the U.S.A [23], Canada [24], and Chile [25], faces the potential drawbacks of mandatory GA fortification, which could lead to overconsumption, especially if FA supplements are also taken [26]. In Europe, foods are currently not fortified. In fact, some authors affirm that efforts to increase folate intake through natural folate-rich foods are unlikely to be effective for health prevention on a population basis [3]. Foods in which we can naturally find folates are green leafy vegetables and citrus fruits, liver, wheat bread, and legumes [27].

As previously stated, FA has a key role in the prevention of birth defects, including NTDs such as spina bifida and other structural malformations. However, the specific mechanism involved is still unknown [28]. In addition, there is suggestive evidence of protection from cardiovascular defects, Down syndrome, limb defects, cleft lip with or without cleft palate, urinary tract anomalies, congenital hydrocephalus, and, more recently, even hearing loss [29,30]. It has been estimated that at least 50% of birth defects can be prevented by improving maternal folate status prior to conception [27,28,31]. Currently, it is recommended that supplementation (400 μg/day) should be started at least 1 month prior to conception and during at least the first 12 weeks of gestation. Moreover, prolonged supplementation throughout all pregnancy is highly recommended considering other maternal–fetal positive effects [29,30], and also in cases of twin pregnancies, chronic illnesses, repeated vomiting, or malabsorption [32]. Supplementation with high FA doses (1–5 mg/day) is recommended for women at high risk: previous pregnancy affected by NTDs, family history with NTDs, twin pregnancies, insulin-dependent diabetes mellitus, obesity (BMI > 30 kg/m^2^), drug/alcohol abuse, or epilepsy, among others [10].

## 2. Objective

Currently, most dietary supplements and virtually all marketed drugs aimed at pregnant women or women planning to become pregnant include folate as FA. However, given that folate is metabolised to 5-MTHF, it has been hypothesised that supplements containing this compound may also be useful in reducing the risk of NTDs [33]. The FDA and the European Food Standards Agency, but not the European Medicines Agency, have already approved products containing a 5-MTHF calcium salt and a 5-MTHF glucosamine salt [34]. Therefore, the objective of the present review is to evaluate, in an evidence-based manner, the clinical efficacy and safety of 5-MTHF and FA as different alternatives to supplementation.

## 3. Materials and Methods

### 3.1. Search Methods

This review is a non-systematic overview of articles published in English and Spanish retrieved from a literature search conducted by means of the following databases: MEDLINE, PubMed, and Scopus, using the following search terms: “folic acid”, “folates”, “5-metyltetrahydrofolate”, “5-MTHF”, “neural tube defects”, “supplementation”, “fortification”, AND “homocysteine” to identify relevant studies published not prior to the year 2000 and up to January 2024. Randomised controlled trials, cohort studies, systematic reviews, and meta-analyses were included, as well as key studies in animal models related to pharmacokinetic studies.

### 3.2. Selection Criteria and Eligibility

Eligible populations comprised women of childbearing age. 

## 4. Results and Discussion

### 4.1. Structure, Metabolism, and Bioavailability of Folates

**Figure 2:**
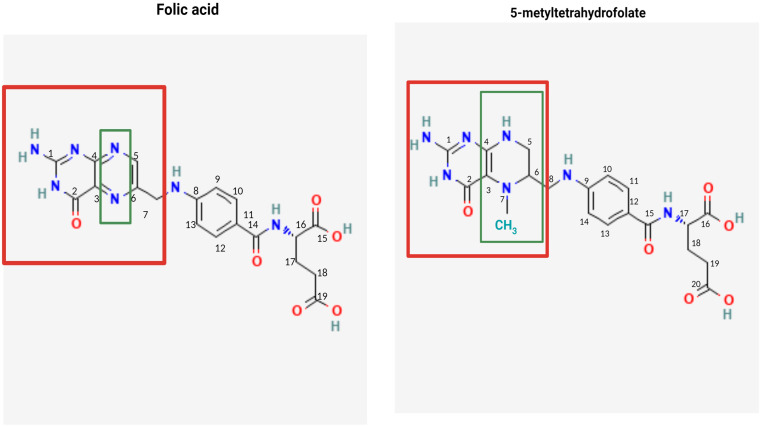
Folic acid and 5-MTHF chemical structures. Created with BioRender.com.

All folates have a common structure formed by a pteridine ring linked by a methylene group to a *p*-aminobenzoic acid, which in turn is linked by an amide bond to one or more glutamate residues [2]. Specifically, the different folates differ in the pteridine ring, which can present several reduced forms and various types of substitutions, and in the *p*-aminobenzoglutamate residue, which can have a variable number of glutamate residues linked in the peptide bond [2] (Figure 2).

What is known as FA, which is the protonated synthetic form, has a structure formed by the union of pteroic acid with a glutamate residue through a peptide bond. This structure is shared by 5-MTHF but with the difference that its pteridine ring has a methyl group in the fifth position, making it a more complex reduced structure than that of FA [2].

Folates, regularly ingested through the diet, are absorbed via intestinal microvilli, and it is essential that they undergo a hydrolysis process catalysed by the enzyme glutamate carboxypeptidase II, which reduces the poly-γ-glutamate tail to only three residues, thereby allowing its entry into the cell cytoplasm [35]. Conversely, FA absorption seems to take place through passive transport in the intestinal mucosa [36].

Folate metabolism is complex since it participates in various metabolic routes involved in the synthesis of compounds of high biological importance, such as purines and pyrimidines, which make up nucleic acids (Figure 1). It also originates from the precursors that participate in the metabolism of methionine, serine, glycine, and histidine, and the methylating agents necessary for normal metabolism and gene regulation [37].

Folate metabolism initially begins with the reduction of the pteridine ring at the eighth nitrogen position of folate, with the consequent formation of DHF. As the reduction in fifth nitrogen continues, THF is formed, this being the active coenzymatic form. Both reactions are catalysed by DHFR. THF can be transformed into 5,10-methyleneTHF by transferring carbons to its 5 and 10 nitrogens in a reaction that can be catalysed by serine hydroxymethyltransferase 1 (SHMT1) or by methylenetetrahydrofolate dehydrogenase 1 (MTHFD1), depending on the source [36]. The 5,10-methyleneTHF resulting from the transformation of THF serves as a source for the synthesis of purines and pyrimidines or can be converted into L-5-MTHF in a reaction catalysed by the methylenetetrahydrofolate reductase (MTHFR) [38]. The resulting L-5-MTHF is the predominant form of folate found in plasma and is used in electron transfer processes in the context of a set of biosynthetic processes, such as in the remethylation of the Hcy into methionine [1,36,38,39] being converted to THF again (Figure 1).

As previously mentioned, FA contains only monoglutamates, while folates in foods have a low absorption rate because they are in the form of polyglutamates, which are incapable of crossing membranes without first being transformed into monoglutamates. Therefore, the greater the number of glutamic acid molecules present in folate molecules, the lower their bioavailability [14]. As a consequence, situations arise in which supplementation with FA is more advisable than obtaining folates through the diet. Furthermore, the reduced form in which folates are found in foods makes them very unstable and easily degraded, with only about 50% being absorbed into the body. FA, on the other hand, is more oxidised and more stable, making its bioavailability close to 100%; however, this can be reduced to 85% if consumed together with food [14].

FA is thermally and chemically stable under proper storage conditions, but precautions should be taken to avoid excessive exposure to light [40,41,42]. On the other hand, 5-MTHF is sensitive to oxygen and temperature; thus, 5-MTHF utility is limited because it is less stable than FA in foods that undergo processing [40,43]. Results about 5-MTHF’s sensitivity to light are controversial [43,44]. Moreover, FA is regulated by pharmacopeial standards to ensure its quality and purity. Both the United States Pharmacopoeia (USP) and the European Pharmacopoeia (Ph. Eur.) provide guidelines and reference standards for FA [45]. Altogether, these data likely underline the fact that FA is the only active substance authorised as a drug and for food fortification.

The bioavailability of dietary folates is variable, depending on the food type and whether they present inhibitors or other unknown factors. As a consequence, to adjust the differences in absorption between folates from foods and FA, the Dietary Folate Equivalent (DFE) is used, which reflects the greater bioavailability from supplements and fortified foods, with each 1 mg of FA equivalent to 1.7 mg of natural folate; that is, FA will be 1.7 times more bioavailable than natural folate from foods [46].

### 4.2. Bioavailability of FA and 5-MTHF

#### 4.2.1. Pharmacokinetic Studies

Owing to the structural differences between the FA and 5-MTHF molecules, it is worth questioning whether there are any differences in their bioavailability. Nevertheless, the studies carried out so far do not show relevant differences in the bioavailability of these compounds.

In 1992, Bhandari and Gregory studied the pharmacokinetic behavior of FA and 5-MTHF in rats, demonstrating identical bioavailability, absorption, tissue distribution, and excretion [47]. It is important to underline that these animals have an intestinal folate transport mechanism identical to that of humans [48]. Prinz-Langehohl et al. studied the comparative bioavailability of equimolar doses of FA or 5-MTHF (equivalent to 400 µg of FA) in a study conducted on 21 healthy women both before and after 10 days of supplementation. The obtained results indicate a slightly greater bioavailability of 5-MTHF at the beginning of the study, but that difference disappeared after 10 days of supplementation [49]. In another study, Pentieva et al. compared the bioavailability of FA and 5-MTHF in healthy men after a single dose of 500 µg of either FA or 5-MTHF, demonstrating a rapid increase in plasma folate concentration, finding total equivalence in the pharmacokinetic parameters analysed (Maximum Plasma Concentration (Cmax), time to Cmax (Tmax), and Area Under the Curve (AUC)) [50]. Likewise, de Meer et al. studied the pharmacokinetic behaviour of FA vs. 5-MTHF in young (<30 years) or middle-aged (>50 years) volunteers at the beginning and after five weeks of supplementation with 400 µg of FA or an amount equimolar of 5-MTHF. In young volunteers, a somewhat lower absorption of FA was observed at the beginning; however, it was somewhat higher at the end of the supplementation period. In the case of the older group, absorption was lower than in the younger one, but no differences were detected either between FA and 5-MTHF or between the beginning and the end of the study [51].

Ohrvik et al. conducted a study in ileostomised patients to compare the bioavailability of FA vs. 5-MTHF administered via fortified bread. Their results indicate a slower absorption in the case of FA, resulting in a lower AUC during the first 12 h, but equivalent considering longer sampling periods. In any case, as the authors indicate, the determination of the actual content of FA or 5-MTHF in bread is different depending on the analytical method used. Comparative analyses were carried out using the lowest FA values, so these results may have a bias, as the authors recognised [52].

Bailey and Ayling [53] examined the pharmacokinetics of FA and 5-MTHF to find a reliable and minimal dose for promptly rescuing folate status prior to critical periods of embryonic development. Serum total folate increased much more rapidly over the first four days in folate-insufficient women given 7.5 mg daily doses of 5-MTHF than in the same of FA. Nearly all women given the former dose every 12 h (in five doses total) almost immediately reached 50 nM serum-total folate, and this level could be maintained by subsequent administration of 400 µg/day of FA. In this case, 5-MTHF enabled repletion of folate stores more quickly and uniformly than FA, but of note, these doses are far beyond the recommended dosages in pregnancy and authorised for 5-MTHF.

In summary, although studies show that 5-MTHF may have a slightly higher bioavailability compared to FA at the start of supplementation, this is then balanced out over the hours/days. Therefore, both compounds would lead to similar bioavailability, regardless of the form of supplementation.

#### 4.2.2. Studies on Erythrocyte Folate Levels

FA erythrocyte levels have been considered an adequate marker of its sufficiency state, with a cut-off level associated with NTD prevention of >906 nmol/L [54]. Several studies have explored the effect of supplementation with FA or 5-MTHF on erythrocyte folate levels, concluding that, at equivalent doses, suitable levels are reached in both cases.

Lamers et al. measured the effect of FA or 5-MTHF supplementation in healthy women aged 19–33 years on erythrocyte folate levels. Three groups of women received 400 µg of FA, equimolar doses, or half the dose of 5-MTHF, for 24 weeks, with determinations performed every 4 weeks. The results indicate that women supplemented with 400 µg of FA or equimolar doses of 5-MTHF achieved significantly higher levels of erythrocyte folate than those receiving half the dose of 5-MTHF. Among those receiving equivalent doses, the 5-MTHF group achieved somewhat higher erythrocyte folate levels than those receiving FA. However, in both cases, the level of 906 nmol/L, considered to prevent NTD development, was widely exceeded, so the therapeutic implications of this difference do not seem relevant [55]. In a similar study, Houghton et al. compared the effectiveness of FA with 5-MTHF in maintaining erythrocyte folate levels during lactation. In this case, a daily dose of 400 µg of FA, equimolar of 5-MTHF, or placebo, was administered from one week postpartum for a total of 16 weeks. Both active groups clearly differed from the placebo group, but no differences in plasma folate levels were detected between active groups. However, erythrocyte folate levels at 16 weeks were slightly lower in the FA group, although high concentrations that surpassed the cut-off of 906 nmol/L were reached in both cases (2193 nmol/L vs. 1891 nmol/L). However, no differences were found in Hcy levels, so it was concluded that both forms are equivalent in increasing and maintaining adequate folate levels [56,57].

#### 4.2.3. Effect on Hcy Levels

Another property of FA supplementation is reducing Hcy by favouring its remethylation to methionine. This, in turn, decreases S-adenosylhomocysteine (SAH), which is responsible for cellular hypomethylation [58]. Hcy is a toxic, non-protein amino acid that contains sulphur and is located in the interconversion pathway of two amino acids: methionine and cysteine [58]. There are many causes of hyperhomocysteinemia, some of which are the result of genetic mutations, nutritional deficiencies (such as PA), systemic pathologies, and environmental and pharmacological toxicity [59]. This can lead to central nervous system deterioration, probably through ischemic mechanisms and synaptic dysfunction, which will produce excitotoxicity [59].

Vast evidence has undoubtedly demonstrated the ability of FA to reduce Hcy levels [60,61,62,63,64,65,66,67,68]. FA supplementation throughout the whole pregnancy has indeed been demonstrated to prevent the increase in Hcy levels taking place in late pregnancy [60,63]. This reduction seems to be also achieved with the administration of a daily dose of 5-MTHF in different population groups, such as women and men of childbearing age, pregnant women, and the elderly, though evidence is more limited. In fact, similar decreases in plasma Hcy concentration between FA and 5-MTHF have been observed [66,67,68].

### 4.3. Clinical Evidence of Dietary Folates, 5-MTHF, and FA

NTDs are major birth defects of the brain and spine that occur early in pregnancy due to inadequate closure of the embryonic neural tube, which can lead to a variety of disabilities or even death. The most common NTDs are anencephaly (an underdeveloped brain and incomplete skull) and spina bifida (incomplete closure of the spinal cord) [69,70]. Though in a study carried out on embryo samples, it was observed that FA intake before pregnancy decreases H3K27 methylation and remodels the chromatin of the Hes1 and Neurog2 promoter genes, which are essential for NTD, so it is clear that FA plays a determining role in the epigenetic regulation of fetal developmental programming [71].

### 4.4. Only FA Has Been Clinically Proven to Be Effective in Preventing NTDs

**Figure 3:**
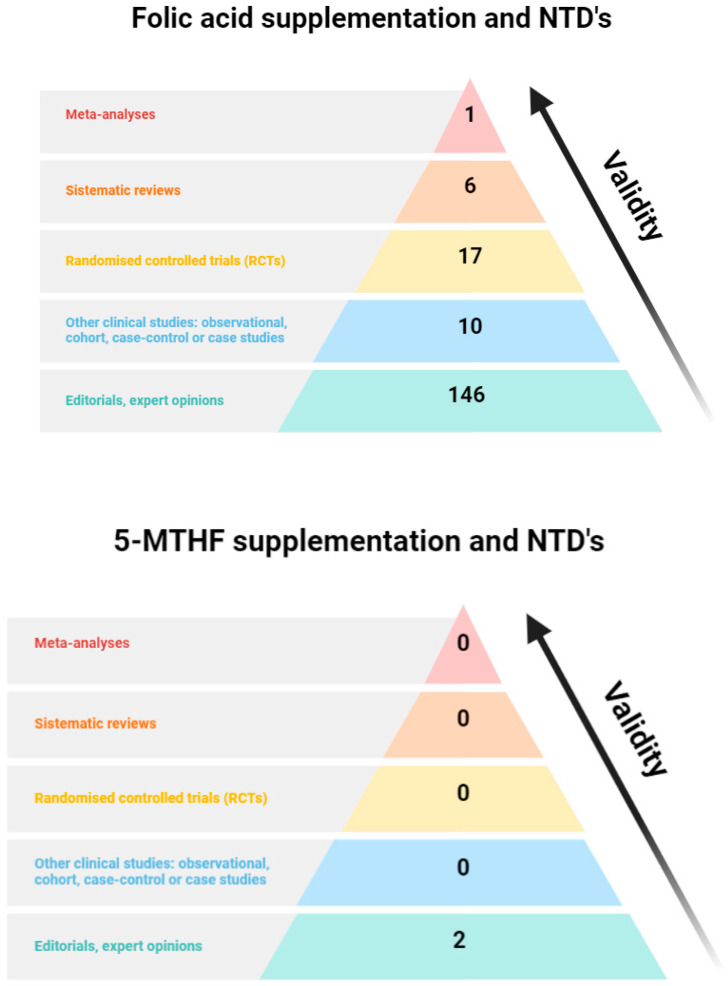
Hierarchy of evidence for FA and 5-MTHF regarding neural tube defects (NTDs). A literature search was conducted in PubMed using the following search terms: ‘folic acid[title] AND neural tube defects[title] AND supplementation’; or ‘(5-MTHF[title] OR methyltetrahydrofolate[title]) AND neural tube defects[title] AND supplementation’. Created with BioRender.com.

Several randomised clinical studies have explored the effect of FA supplementation in preventing the occurrence of NTDs (Figure 3). FA supplementation during pregnancy has been very firmly established as crucial for reducing NTD risk in infants, with most studies involving a daily dose of 400 µg [72]. In this regard, a recent Cochrane review reviewed all clinical studies carried out with adequate quality, concluding that the evidence of the effectiveness of peri–postconceptional FA in the prevention of NTDs is high according to the GRADE (Grading of Recommendations, Assessment, Development, and Evaluations) Working Group. In fact, rates of 69% and 66% risk reductions in NTD occurrence and recurrence, respectively, were obtained [73]. Recent data have further confirmed these results (OR = 0.56), particularly marked when supplementation started 3 months before conception [30]. As a result of this efficacy, policies on FA supplementation during pregnancy are recognised as one of the key factors associated with the reduction in NTDs observed in the last decades in many countries [74,75]. Oppositely, to our knowledge, there are no published clinical studies that measure the effect of 5-MTHF supplementation in NTD prevention (Figure 3), concluding that, although 5-MTHF has been proposed as an alternative to FA, “more research is needed in the use of other type of supplements such as 5-MTHF” [73]. In fact, experts from the National Center on Birth Defects and Developmental Disabilities (CDC) recently indicated that “randomised controlled trials would have to be conducted to determine effectiveness, timing, dosage, stability, and safety in order for 5-MTHF (or a synthetic equivalent) to be recommended” [1].

#### Far beyond NTDs: Clinical Evidence of FA on Other Maternal and Fetal Outcomes

Likewise, a cause-and-effect relationship has been established by EFSA between folate and blood formation, normal Hcy metabolism, immune system function, cell division, and maternal tissue growth during pregnancy [76]. Effectiveness is known for the prevention of common maternal health problems such as megaloblastic anaemia, infections, preeclampsia, uterine haemorrhage, abrupt placental abruption, intrauterine growth retardation, and prematurity [29]. A meta-analysis with 3839 pregnancies found a significant reduction in the incidence of megaloblastic anaemia with FA supplementation (OR = 0.21) [77].

There are studies that have shown that FA intake reduces the frequency of heart defects, lip and palate clefts, urinary tract defects, anterior wall closure, and limb defects. A meta-analysis of studies using a multivitamin supplement containing FA demonstrated a reduction in the risk of heart (OR = 0.78), cleft lip or palate (OR = 0.76), and limb defects (OR = 0.48) [78]. Moreover, a very recent publication from Zhou Q et al. showed that the odds of birth defects, including congenital heart disease and clefts, were significantly lower among women under FA supplementation over 3 months before pregnancy (OR = 0.63 and R = 0.47, respectively, *p* < 0.05) [29,30].

Furthermore, several studies and meta-analyses have shown that FA supplementation significantly reduces the risk of small for gestational age at birth (ORs 0.72 to 0.9) [29,79]. Specifically, in Guo et al.’s study [80], an additional improvement in this parameter was observed when FA was supplemented throughout pregnancy compared to only in the first trimester, and a reduction in low birth weight was also observed [80]. On the other hand, controversial results were found in studies that evaluated the reduction of preeclampsia risk. Hence, a systematic review concluded that the relationship between FA supplementation and the prevention of preeclampsia is uncertain [77,81], while another found positive results in the prevention of this pathology [82]. Finally, some studies evaluated the cognitive parameters of children whose mothers received FA supplementation. Cognitive improvements were observed in children whose mothers received supplementation during the whole pregnancy, concluding a positive association with continued FA supplementation beyond the first trimester of pregnancy [83,84].

Of note is that at present, there is no solid positive evidence of the beneficial effects of 5-MTHF, at supplementation doses, in the improvement of these conditions [85,86].

### 4.5. Effect of Genetic Background on the Effectiveness of FA

Some polymorphisms of genes encoding enzymes implicated in folate metabolism are reported to have an impact on folate status and health consequences [87]. The highest impact has been reported for the *677C->T* polymorphism of the gene encoding the MTHFR enzyme. MTHFR converts 5,10-methylene-THF to 5-methyl-THF, providing one-carbon units for the methylation cycle. Therefore, a proportion of the population would theoretically metabolise folate more slowly because of defects in the MTHFR enzyme, which is the rate-limiting enzyme in the methylation cycle. Of note, given the cyclic nature of folate metabolism (Figure 1), any folate vitamer could theoretically be impacted by this polymorphism.

Homozygosity for the *T* allele is linked with decreased enzyme activity (up to 70% lower). However, even in basal conditions (i.e., non-supplemented), the difference in the capacity to metabolise folate due to the different variants of the *MTHFR* gene is small, with an approximately 13–25% difference between the genotype of maximum (*CC*) and minimum (*TT*) activity in serum folate and plasma total Hcy concentrations [88,89]. Under supplementation conditions, the administration of 400 µg of FA has been shown to eliminate these differences [90]. It should be noted that routine screening for the *MTHFR C677T* variant is not recommended by the American College of Obstetricians and Gynecologists [91].

Berry et al. studied the effect on NTD prevention in a total of 130,142 deliveries of women who took a 400 µg FA supplement during the periconceptional stage, and the first trimester of pregnancy compared to a group of 117,689 non-supplemented women. This study was conducted in different regions of China, a country with genotypically different ethnicities. In the population of Northern China, the prevalence of the *TT* (low-activity) genotype is much higher than in the South, resulting in non-supplemented women having a relative risk of NTDs that is eight times higher in the North than in the South. However, in women who took FA, there was a significant decrease in the rate of NTDs in both groups, resulting in similar rates in both populations. Significantly, considering only women who had therapeutic compliance greater than 80%, the decrease was more pronounced, reducing the incidence of NTDs to identical levels in both cases [92]. In a subanalysis of this previous study, Crider et al. directly studied the influence of women’s genotype on erythrocyte folate levels. In total, they analyzed 932 women, of which 35.1% were *TT* homozygotes (low activity), 17.4% were *CC* homozygotes (high activity), and the remaining 47.5% were *CT* heterozygotes. The level of erythrocyte folate was associated with the genotype, being higher in those with the *CC* genotype than in the *TT*. Despite this association, in women supplemented with 400 µg/day of FA, the target value of 906 nmol/L was reached (mean value, 927 nmol/L). It is important to additionally indicate that results reported in the main study demonstrated the same level of NTD reduction in populations with different *MTHFR* genotypes, proving that the erythrocyte folate levels achieved were clinically adequate [93]. In a similar vein, Zhou Q et al. showed data from a prospective cohort study, also in China, with the same prevalence of polymorphisms as reported in Berry et al. In this study, it was observed that regardless of the variant, the odds of birth defects, including NTDs, were significantly lower among women under FA supplementation over 3 months before pregnancy (*p* < 0.001) [30].

Regarding Hcy, total plasma Hcy levels decreased significantly in both subjects receiving 1 mg FA or 5-MTHF over time; however, no differences in total Hcy levels nor abortion rates were observed between the groups. The results did not support any beneficial effect of 5-MTHF vs. folate supplementation in women with recurrent abortion in any *MTHFR* polymorphism [94]. Altogether, 400 μg of FA effectively decreases the incidence of NTDs in women, regardless of MTHFR polymorphisms. In view of the evidence, scientific societies, including the CDC, urge all women of childbearing age who may become pregnant to consume 400 µg/day of FA, including those with an MTHFR C677T variant [8].

### 4.6. Safety of Dietary Folates, 5-MTHF, and FA

Although FA has a water-soluble nature and rarely produces toxicity as excessive amounts tend to be eliminated through urine, and it does not accumulate in tissues, adverse reactions might occur at high amounts, such as diarrhoea, nausea, abdominal cramps, bloating and gas, rash, insomnia, zinc deficiency, psychotic behaviour, seizures, bitter taste in the mouth, hyperactivity, irritability, or excitability [95,96]. Of note, these adverse reactions are reported as rare or at unknown frequency, even for drugs containing 5 mg of FA [97]. In terms of FA and cancer, many large studies and meta-analyses have not found any relationship between FA supplementation and developing additional colorectal adenomas [98]. Oppositely, to our knowledge, 5-MTHF safety has not yet been established, and properly sample-sized, phase II–III clinical trials in pregnant women are not available for this metabolite.

#### Excessive FA Intake and Masking Vitamin-B12 Deficiency

Vitamin-B12 deficiency can cause neurological complications to the brain, spinal cord, and nervous system, which, if untreated, may be irreversible. This hypothetical situation is particularly concerning for older adults, who are more prone to this vitamin deficiency [19].

Amongst the transformations of the folate cycle, a portion is converted to 5-MTHF. This form, until it is used in DNA synthesis and cell division, must be reconverted to THF, but this conversion requires vitamin B12 (Figure 1). Therefore, in situations of vitamin-B12 deficiency, much of the folate is trapped in the form of 5-MTHF and does not participate in DNA synthesis. This situation is called the “folate trap” or “methyl trap”, leading to insufficient DNA production, further leading to the condition clinically known as megaloblastic anaemia [99]. The administration of high doses of FA can activate the synthesis of purines and pyrimidines, correcting the symptoms of this anaemia. However, if it is accompanied by a vitamin-B12 deficiency, long-term treatment with high FA doses can mask this deficiency and would potentially aggravate the symptoms of vitamin-B12 deficiency [100].

However, it has been proposed that 5-MTHF supplementation may have a lower risk of hiding haematological symptoms due to vitamin-B12 deficiency in patients with megaloblastic anaemia since, unlike FA, the first step in the folate cycle for 5-MTHF requires vitamin B12 (converted into THF). Therefore, in the absence of B12, the synthesis of purines and pyrimidines is not activated [101], and thus, masking the haematological symptoms of a vitamin-B12 deficiency could not occur [44].

In any case, it should be noted that most pregnancy supplements contain vitamin B12 at the recommended pregnancy daily allowance, preventing vitamin-B12 deficiency, and low doses of FA (400 µg); thus, this risk of vitamin-B12 masking is considered of minimal concern.

### 4.7. Unmetabolised FA

Circulating non-used FA in the body is called “unmetabolized folic acid” (UMFA). Importantly, UMFA has been identified in 95% of people, irrespective of intake and supplement use [102]. Likewise, it has also been observed that supplementation with high doses (well above the recommended ones) of FA can lead to saturation/inhibition of the DHFR enzyme, leading to an accumulation of UMFA and, as a consequence, UMFA syndrome [103]. This syndrome is characterised by the competition of unmetabolised FA with 5-MTHF for the folate transporter (SLC19A1) and the folate receptor (FolR1), thus preventing 5-MTHF from participating in metabolic cycles, affecting the recycling of Hcy [104,105]. This effect is suggested not to occur, by definition, after the administration of 5-MTHF [106]. The latter hypothesis, however, needs to be scientifically evidenced and proven.

In the work by Tam et al. [107], this increase in UMFA was transient, occurring after 12 weeks of treatment but returning to basal levels at 30 weeks in women of childbearing age. There is no clear information regarding the meaning and clinical consequences of UMFA, although it has been associated with different pathologies. Thus, UMFA has been attributed to a reduction in the activity of NK cells, decreasing the ability of the immune system to kill malignant or pre-malignant cells, suggesting a relationship between UMFA and cancer.

In this regard, there is only one published study reporting lower NK cell activity in women aged 60 to 75 years with high UMFA levels, but this supposed association is not found in women between 50–59 years old [108]. The most logical explanation for this apparent contradiction is that both phenomena are not associated. There is a known reduction in the activity of the immune system in older women, and concomitantly, but not in association, there is a reduction in liver function, resulting in a lower metabolism of FA. Therefore, there appears to be some temporal association between elevated UMFA levels and NK cell activity but no causal association. Some further work is beginning to be reported [109], and therefore, we cannot state that UMFA is safe.

An association of UMFA with colon cancer has been contemplated due to an increase in the number of cases that coincide with the initiation of FA fortification (both in 1996). However, this association would imply an immediate effect of FA intake on the incidence of colon cancer, which is implausible [110]. Subsequent larger studies in the post-fortification stage have not shown any increased risk of colorectal or pancreatic cancer [111,112]. Likewise, a retrospective case-control study conducted on a large population before fortification (part of the study population took FA supplements) has found no association between UMFA levels and colorectal cancer [18].

Altogether, despite years of study, circulating UMFA has not been confirmed to have adverse effects. In this regard, experts from the National Centre on Birth Defects and Developmental Disabilities from the CDC recently indicated that “it is not necessary to replace a known effective intervention with one that does not have established effectiveness” [1].

To conclude, 5-MTHF supplementation has been proposed to have some theoretical advantages over FA elsewhere [44]: higher bioavailability, direct entrance to the folate cycle (no need to be transformed by DHFR, no risk of DHFR saturation and accumulation of UMFA in the blood), no influence of MTHFR polymorphisms, and fragility to UV-A radiation. Overall, the above-reviewed studies indicate that FA supplementation produces plasma and erythrocyte folate levels similar to those produced with 5-MTHF, regardless of the MTHFR variant. No confirmed health implications have been described for UMFA, and more importantly, only FA has been clinically proven to be effective in preventing NTDs and other maternal–fetal outcomes. There are no published clinical studies demonstrating the effect of 5-MTHF supplementation on the prevention of NTDs [1].

## 5. Conclusions

The efficacy and safety of FA in the recommended doses for the prevention of neural tube defects have been well supported in many different clinical studies for decades. However, despite the potential value of 5-MTHF as an alternative to FA, due to its metabolic properties, clinical studies would be urgently needed to support the efficacy, dosages, timings, and/or safety of its use as a supplement. The clearly different clinical development between both metabolites likely underlies the worldwide use, international recommendations, and long-lasting differential approval of FA as a drug compared with 5-MTHF.

## Data Availability

The data presented in this study are available on request from the corresponding author.
